# Non-Alcoholic Fatty Liver Disease and Metabolic Syndrome after Liver Transplant

**DOI:** 10.3390/ijms17040490

**Published:** 2016-04-02

**Authors:** Stefano Gitto, Erica Villa

**Affiliations:** Department of Gastroenterology, Azienda Ospedaliero-Universitaria and University of Modena and Reggio Emilia, Via del Pozzo 1, 41124 Modena, Italy; stefano.gitto@studio.unibo.it

**Keywords:** liver transplant, multifactorial disease, metabolic syndrome, non-alcoholic fatty liver disease, non-alcoholic steatohepatitis

## Abstract

Liver transplant is the unique curative therapy for patients with acute liver failure or end-stage liver disease, with or without hepatocellular carcinoma. Increase of body weight, onset of insulin resistance and drug-induced alterations of metabolism are reported in liver transplant recipients. In this context, post-transplant diabetes mellitus, hyperlipidemia, and arterial hypertension can be often diagnosed. Multifactorial illnesses occurring in the post-transplant period represent significant causes of morbidity and mortality. This is especially true for metabolic syndrome. Non-alcoholic steatosis and steatohepatitis are hepatic manifestations of metabolic syndrome and after liver transplant both recurrent and *de novo* steatosis can be found. Usually, post-transplant steatosis shows an indolent outcome with few cases of fibrosis progression. However, in the post-transplant setting, both metabolic syndrome and steatosis might play a key role in the stratification of morbidity and mortality risk, being commonly associated with cardiovascular disease. The single components of metabolic syndrome can be treated with targeted drugs while lifestyle intervention is the only reasonable therapeutic approach for transplant patients with non-alcoholic steatosis or steatohepatitis.

## 1. Introduction

Liver transplant (LT) represents the curative treatment for patients with acute liver failure, end-stage liver disease and/or non-resectable hepatocellular carcinoma worldwide. After surgery, transplanted patients often develop an increase of body weight, insulin resistance (IR) and metabolic alterations [1]. Multifactorial disease such as diabetes mellitus (DM), hyperlipidemia and arterial hypertension are common complications after LT, all negatively affecting quality of life, morbidity and mortality [1]. Consolidated immunosuppressant drugs such as corticosteroids, calcineurin inhibitors (CNIs) (cyclosporine (CSA) and tacrolimus (TAC)) and mammalian target of rapamycin inhibitors (mTORs) (such as sirolimus (SIR)) play a key role in the metabolic balance, favoring hyperglycemia, arterial hypertension and hyperlipidemia [2]. In this context, a significant amount of transplanted patients fulfill the criteria of metabolic syndrome (MS) which is strongly associated with an increased cardiovascular risk [1]. Since non-alcoholic fatty liver disease (NAFLD) and non-alcoholic steatohepatitis (NASH) are considered the liver expression of MS, it is not surprising that both recurrent and *de novo* NAFLD/NASH can be found after LT [3]. Although post-LT steatosis shows an indolent outcome in terms of fibrosis progression, NAFLD/NASH should be considered for the stratification of morbidity and mortality risk of transplant patients. Notably, cardiovascular disease represents the major cause of death unrelated to liver disease and the third most common cause of mortality among transplant patients, accounting for 12%–16% of deaths. Today, targeted drugs for MS and NAFLD/NASH do not exist. Clinicians can use specific drugs against the single components of MS while a strong improvement of behavior in terms of diet and aerobic exercise is the only reasonable approach for recurrent or *de novo* NAFLD/NASH [1].

This review article focuses on the current literature regarding the main metabolic diseases affecting transplanted patients, the clinical impact of post-LT MS and NAFLD/NASH and, finally, the feasible therapeutic strategies.

## 2. Multifactorial Disease after Liver Transplant

The majority of transplant patients develop a rise in body weight after surgery. The highest weight increase occurs after the first six months and at one and three years from LT, and the median weight gain is 5.1 and 9.5 kg, respectively. Notably, at one and three years, 24% and 31% of transplant patients become obese [4]. However, the above-cited authors [4] reported that the vast part of enrolled patients were also obese before LT. Considering only patients who were not obese at the time of surgery, 15.5% at one year and 26.3% at three years had a body mass index (BMI) >30 [4]. In a further study, 23 patients were followed for nine months after LT. At the end of the study, 87 of the subjects were overweight or obese with a significant increase in fat mass and a minor improvement in lean mass [5]. Another study [6] showed progressive weight gain in the first year after LT, with one-third of patients becoming obese at the end of observation. Considering a follow-up of four years, overweight and obesity were found in 58% and 21% of cases and high BMI before LT was the main risk factor of post-LT obesity [7].

In this context, DM, hyperlipidemia and arterial hypertension can be often diagnosed after LT [1] (see Table 1).

Post-LT DM is associated with more significant morbidity with respect to pre-LT disease, determining an increased risk of post-operative infection and cardiovascular events [8,18]. The incidence of post-LT DM ranges from 10% to 64% [9]. Ahn *et al.* [19] showed that among 74 patients transplanted with post-LT DM, post-LT DM was transient in 56.8%, while in the others it was persistent. Although the underlying mechanisms are not yet clear, the main risk factors for the onset of post-LT DM are the following: male gender, high pre-LT BMI, positive family history, hepatitis C virus infection, older age, high dosage of immunosuppressant drugs and rapamycin gene polymorphisms [8]. A meta-analysis confirmed that male gender, high pre-LT BMI and positive family history are predictive of post-LT DM development [10]. Transcription factor 7-like 2 (TCF7L2) protein regulates cell proliferation and differentiation modifying the insulin secretion [20]. Notably, it was reported that polymorphisms of the *TCF7L2* gene in LT donors are another independent risk factor of post-LT DM [11].

Among transplanted patients, a percentage ranging from 45% to 69% develops hyperlipidemia, which is a significant risk factor for cardiovascular morbidity and mortality [12]. Increased nutrient intake, older age, body weight, presence of DM, renal impairment, immunosuppressive drugs, such as steroids, CSA, TAC, and SIR, are risk factors for post-LT hyperlipidemia [13,14]. Interestingly, the polymorphism of the low-density lipoprotein receptor gene in the donor may facilitate the development of hyperlipidemia in the recipient [15].

Arterial hypertension, an uncommon feature in subjects with chronic liver disease, arises in 50%–100% of patients after LT [9,16]. Post-LT hypertension usually develops in the first six months after LT as a consequence of systemic vasoconstriction, elevation in plasma endothelin-1 concentrations, and increased arterial stiffness [21]. Occurrence of post-LT hypertension is favored by obesity and older age and is often associated with impaired glycemia. Moreover, it is well known that both CNIs and corticosteroids have negative effects on pressure control [17].

## 3. Metabolic Impact of Immunosuppressant Drugs

It is well known that immunosuppressive agents might exert negative metabolic effects [22] (see Table 2).

Corticosteroids represent a key component of the immunosuppressant protocol in the first months after LT but are also necessary in the long-term management of patients transplanted for autoimmune or cholestatic liver disease. Corticosteroids show dose-related metabolic side effects. They increase appetite and fat depositions, drop fat oxidation, and lead to increased proteolysis and reduced protein synthesis [23,24]. Moreover, high doses of corticosteroids determine the rise of both IR and gluconeogenesis [25]. Corticosteroids also negatively alter lipid metabolism and steroid-free protocols might lead to a significant decrease in hypertriglyceridemia [34]. Corticosteroids also influence mineralocorticoid metabolism, causing sodium retention. Interestingly, steroids directly correlate with NAFLD/NASH occurrence in liver allografts [35].

CNIs may negatively affect energy metabolism and muscle mass [26] and CSA represents an independent predictor of post-LT weight gain [36]. Through a meta-analysis including 10 studies, Li *et al.* [10] demonstrated that TAC is an independent risk factor for post-LT DM. Regarding lipid metabolism, CSA has a more negative effect in comparison with TAC. The incidence of hyperlipidemia is higher in patients treated with CSA than with TAC (14% *versus* 5% and 49% *versus* 17%) [27,28]. CNIs also favor the onset of arterial hypertension determining arterial vasoconstriction. Among CNIs, TAC seems to have a lesser impact on arterial pressure in comparison to CSA, but data are not conclusive [29,30]. As expected, minimizing the use of CNIs improves their metabolic profile and, consequently, the long-term outcome of patients [37,38].

SIR increases triglyceride production, being the most dangerous immunosuppressant in terms of lipid alteration. Among patients treated with SIR, 55% develop hyperlipidemia [31]. In addition, SIR alters the insulin signaling pathway [31] and negatively affects muscle mass status [32]. Recently, Zimmermann *et al.* [33] conducted a study involving 92 transplant patients, reporting that patients treated with mTORs were at higher risk of hyperlipidemia and glycemic alteration with respect to patients under CNIs.

## 4. Metabolic Syndrome after Transplant

The definition of MS includes a combination of at least three of the following factors: arterial hypertension, IR, hypertriglyceridemia, low high-density lipoprotein and obesity [39]. In the post-LT period, MS can be found in 50%–60% of patients. MS represents a relevant risk factor for atherosclerosis and cardiovascular disease, which are the main causes of post-LT morbidity and mortality [39]. Interestingly, the prevalence of post-LT MS is about twice that of the general North American population [40]. Older age, obesity, pre-LT DM, genetic polymorphisms in the living donor and the use of high-dosage immunosuppressive drugs are risk factors for post-LT MS [9]. Sprinzl *et al.* [41] analyzed a cohort of 170 transplant patients with a follow-up of two years. The authors showed that *de novo* MS was present in one-third of patients and glycosylated hemoglobin ≥5% and arterial hypertension were independent risk factors for it. Moreover, the authors demonstrated a negative dose-dependent role for steroids. It was also confirmed that in the post-LT period, MS could be considered as a link toward NAFLD/NASH. Interestingly, it was reported that changes in intestinal microbiota might also play a relevant role in the development of MS after LT [42]. Fussner *et al.* [43] retrospectively analyzed 455 consecutive LT recipients with a long follow-up (8–12 years), suggesting that increased BMI was a strong predictor of MS at one year from the LT. Consequently, the authors suggested that preventing weight gain in the early months after LT might decrease the probability of MS. However, the authors suggested that older age, post-LT DM, prior family history of cardiovascular disease, altered serum troponin, but not MS, were independent predictors of cardiovascular events. It has to be underlined that specific treatments for MS are not yet available, while the only feasible way to manage it is to treat its single components [44].

## 5. Post-Transplant Non-Alcoholic Fatty Liver Disease

In the pre-LT period, NAFLD and NASH represent the liver expression of altered metabolic status being associated in a large number of cases to IR, dyslipidemia and obesity. Considering the significant prevalence of metabolic diseases after LT, it is clear why both recurrent and *de novo* NAFLD/NASH can be found in transplant patients [41].

Burra *et al.* [45] reported that NASH recurrence ranges from 20% to 40%, this wide variability depending on the methodology used for the diagnosis. Notably, in the majority of cases the outcome of recurrent NAFLD/NASH is harmless, without an evolution toward cirrhosis [46]. Nevertheless, patients with recurrent NAFLD/NASH more frequently show cardiovascular disease and worse infection-related morbidity and mortality. This is evident considering that the recurrence of NASH is associated with DM, weight gain, and dyslipidemia [47]. Interestingly, genetic predisposition might play a role in the recurrence of NAFLD and NASH. The presence of the rs738409-G allele of the Patatin-like phospholipase in the LT recipients represents an independent risk factor for post-LT obesity, DM and steatosis [48,49].

The leading risk factors for the development of *de novo* NAFLD/NASH are the following: obesity, hyperlipidemia, DM, arterial hypertension, TAC-based immunosuppression, pre-LT alcoholic cirrhosis and liver graft steatosis [50]. Sprinzl *et al.* [41] analyzed the association between MS and post-LT NAFLD/NASH. Mixed vesicular steatosis was observed in 34.1% of patients. Hepatic steatosis was mild, moderate, and severe in 16.5%, 7.1%, and 2.9% of cases. Among patients with MS and steatosis, NASH was diagnosed only in 5.4% of patients, confirming that post-LT metabolic liver disease might be relevant not as a primary liver disease but as an indicator of cardiovascular risk. Remarkably, NAFLD/NASH patients showed higher triglyceride levels, elevated uric acid and higher BMI with respect to patients with MS but without liver disease. The authors demonstrated that obesity and dyslipidemia but not arterial hypertension and DM favored the onset of NAFLD/NASH among transplanted patients with MS. Another interesting assumption was that a BMI greater than 28.9 was the only specific risk factor for histological NASH. Mikolasevic *et al.* [51] identified the association between NAFLD/NASH and the development of post-LT cardiovascular and chronic kidney disease. Consequently, according to these authors, diagnosing NAFLD/NASH in the post-LT period might improve the stratification of cardiovascular and kidney damage risk.

## 6. Therapeutic Approach against Post-Transplant Dysmetabolism

The knowledge of pathogenesis is central for understanding the rationale of the therapeutic approach against MS and NAFLD/NASH. The onset of IR represents a true turning point. In fact, IR determines a status of chronic inflammation that favors the other metabolic alterations [52]. The molecular basis of IR depends on both genetic and non-genetic mechanisms. IR determines a chain of events involving inflammation, hypercoagulability, and atherogenesis. Notably, IR occurs firstly in the vascular structures, and this is one of the main reasons for its association with cardiovascular disease [53]. Regarding the NAFLD/NASH, the latest proposed model is the “multiparallel hits” [54]. According to this hypothesis, many events happen in parallel, and all are potential therapeutic targets. The main pathological characters are IR, oxidative stress, adipose and pancreatic tissues, altered lipid metabolism, bile acids, gut microbiota, and bacterial endotoxins.

As we reported, transplant patients often develop IR and an increase in body weight [1]. Interestingly, Kouz *et al.* [55] demonstrated that in patients transplanted for NASH-cirrhosis, most of the weight gain occurs in the first year after LT, while the increase of the weight is more progressive in subjects with a different etiology. However, regardless of the kind of pre-LT liver disease, after LT a relevant increase in dietary intake can be found, especially in patients with pre-LT severe dietary restrictions, gastrointestinal symptoms or anorexia. In detail, from the pre-LT period to one year after LT, calories rise from 27 to 32 kCal/kg and proteins from 0.8 to 1.3 g/kg per day [56]. Richardson *et al.* [5] showed that in overweight or obese transplant patients, more significant energy intake, higher consumption of both proteins and carbohydrates and doubled intake of fat can be found with respect to the pre-LT period.

The feasible pharmacological tools for treating the single metabolic disease, associated or not with NAFLD/NASH, should be used with caution for the possible drug-drug interactions [57]. Notably, a single drug for post-LT MS is not available. Based on these considerations, the main intervention after surgery should be a strong lifestyle control for both prevention and treatment of MS. However, the only randomized trial of exercise and dietary counseling after LT published in 2006 did not show a real advantage with this approach [58]. In this study, 151 liver transplant patients, randomized into exercise and dietary counseling or usual care, showed a similar increase in body weight, fat mass and lean mass. It should be underlined that full adherence to exercise and nutrition was obtained only in 37% of subjects.

Many drugs have been proposed for the treatment of NAFLD/NASH, but lifestyle intervention should be the first-line therapy. In particular, lifestyle modification is the standard of care according to the Italian, European, Asian-Pacific and North American guidelines [59,60,61,62]. The main targets for the usefulness evaluation should be a weight loss of 7% and 150 min/week of physical activity [63,64]. In particular, a weight loss of 7% has been seen to significantly decrease fat accumulation and reduce necroinflammation in non-transplanted patients with NAFLD/NASH [63]. Markedly, aerobic and resistance physical activity have an independent positive effect in decreasing fat in the liver, regardless of the weight loss [65,66]. Furthermore, clinicians should take into account that the physical activity *per se* improves cardio-respiratory fitness [67,68]. Vitamin E and pioglitazone represent the first-line pharmacological options. Both vitamin E and pioglitazone improve fat accumulation and liver inflammation. However, the use of vitamin E is limited to patients without DM and it has no clear effects on fibrosis. On the other hand, pioglitazone shows a negative impact on patients’ weight. In addition, the long-term safety of these drugs is uncertain. Many other drugs such as metformin, ursodeoxycholic acid, statins, pentoxifylline, and orlistat have been tested in pilot studies or randomized clinical trials with few results in terms of efficacy. Telmisartan, a safe antihypertensive drug, is an emerging drug with an interesting preliminary effect on NAFLD/NASH. It seems to have a positive impact on IR, liver steatosis, inflammation, and fibrosis [52]. As recently reported in a review article by Lassailly *et al.* [69], many other drugs are in progress for the treatment of NAFLD/NASH, including obeticholic acid, liraglutide and elafibranor. Authors also suggest that bariatric surgery may be successful in well-selected obese patients with NAFLD/NASH.

Concerning the transplanted patient, none of the cited therapeutic options have been validated.

## 7. Conclusions

Starting in the first months after surgery, transplant patients tend to develop overweight or obesity, IR and, consequently, multifactorial diseases. Consequently, a high prevalence of multifactorial disease such as DM, hyperlipidemia and arterial hypertension can be found. All these metabolic features negatively influence the outcome of transplant patients in terms of quality of life, morbidity and mortality.

All the main immunosuppressant drugs, such as corticosteroids, CSA, TAC and SIR, favor the onset of metabolic alterations. Corticosteroids are surely very important in the first months after LT but also in the long-term in selected cases. They lead to weight gain and fat accumulation negatively affecting lipid, glycemic and pressure profiles. Moreover, they directly increase the risk of steatosis development. CNIs have a negative metabolic impact since they increase weight gain and reduce muscle mass. TAC seems to be superior compared to CSA concerning the metabolic risk in terms of the alteration of lipid and arterial pressure. It should be the first choice among CNIs. SIR is the immunosuppressant with the worst lipid profile. Moreover, SIR shows a worse glycemic profile with respect to CNIs and has a negative effect on the muscle mass status. The choice of immunosuppressant is central and related to many aspects and evaluations such as the cardiovascular and renal risks. In general, one of the main aims of clinicians should be to minimize the dosage of immunosuppressants. This last assumption is true especially in the long-term period and in patients with pre-LT etiology different from autoimmune or cholestatic disease and without a history of graft rejection.

The presence of criteria for MS is frequent in the post-LT period and represents the main indicator of cardiovascular-related morbidity and mortality. NAFLD and its progressive form, represented by NASH, can be considered the liver expression of MS. Indeed, both recurrent and *de novo* NAFLD can be diagnosed in transplanted patients. The hepatic outcome of steatosis after surgery is generally not very aggressive, with few percentages of advanced fibrosis, in comparison with the pre-LT phase. However, together with MS, steatosis is a relevant indicator of increased cardiovascular risk. This assumption is important if we consider that cardiovascular disease is found in 10.6%, 20.7%, and 30.3% of recipients at one, five, and eight years from the LT [43]. Interestingly, post-LT NAFLD/NASH is also associated with an increased risk of infections and renal injury.

Clinicians might definitely use the diagnosis of NAFLD in the post-LT period as an indicator of increased cardiovascular and renal risk. Transplant patients with a first diagnosis of NAFLD should be closely monitored regarding peripheral atherosclerotic signs and kidney function. In this direction, the development of diagnostic algorithms with the use non-invasive tools is warranted. Karlas *et al.* [70] demonstrated that modern non-invasive liver graft assessments such as hepatic ultrasound and transient elastography might be able to properly detect both steatosis and graft fibrosis.

Specific therapeutic options against post-LT MS or NAFLD are not available. Targeted pharmacological tools can be used for each component of MS. So far, a strong behavioral change in terms of diet and aerobic exercise is the only reasonable approach for transplant patients for both primary and secondary care. Transplant patients should be educated starting from the first weeks after surgery for preventing the development of multifactorial diseases, MS and metabolic liver illness. A well-done stratification of the cardiovascular risk should be developed as soon as possible after LT. In the next years, the genetic study of recipients and donors might improve the quality of organ allocation, decreasing the metabolic complications after LT.

## Figures and Tables

**Table 1:** Multifactorial conditions affecting transplant patients.

Disease	Incidence	Risk Factors	References
Diabetes mellitus	10%–64%	Male gender, high pre-LT BMI, family history, hepatitis C, older age, immunosuppressants, rapamycin gene polymorphisms, *TCF7L2* gene polymorphisms (donor)	[8,9,10,11]
Hyperlipidemia	45%–69%	Diet, older age, high BMI, DM, renal impairment, immunosuppressants, low-density lipoprotein receptor gene polymorphism (donor)	[12,13,14,15]
Arterial hypertension	50%–100%	Obesity, older age, impaired glycemia, immunosuppressants	[9,16,17]

LT: liver transplant; BMI, body mass index; TCF7L2, Transcription factor 7-like 2; DM, diabetes mellitus.

**Table 2:** Most used immunosuppressant drugs and main metabolic side effects.

Drug	Side Effects	References
Corticosteroids	Increased fat depositions, decreased fat oxidation, increased proteolysis, reduced protein synthesis, IR, hyperlipidemia, sodium retention, NAFLD	[23,24,25]
CSA	Decreased energy metabolism and muscle mass, weight gain, hyperlipidemia, arterial hypertension	[26,27,28,29,30]
TAC	DM, hyperlipidemia, arterial hypertension	[10,27,28,29,30]
SIR	Decreased muscle mass, hyperlipidemia, glycemic alteration	[31,32,33]

CSA: cyclosporine; TAC: tacrolimus; SIR: sirolimus; IR, insulin resistance; NAFLD, non-alcoholic fatty liver disease; DM, diabetes mellitus.

## Abbreviations

LT	Liver Transplant
IR	insulin resistance
DM	Diabetes mellitus
CNIs	calcineurin inhibitors
CSA	cyclosporine
TAC	tacrolimus
mTORs	mammalian target of rapamycin inhibitors
SIR	sirolimus
MS	Metabolic Syndrome
NAFLD	Non-alcoholic fatty liver disease
NASH	Non-alcoholic steatohepatitis
BMI	body mass index
TCF7L2	Transcription factor 7-like 2
